# Facile fabrication and mechanistic understanding of a transparent reversible superhydrophobic – superhydrophilic surface

**DOI:** 10.1038/s41598-018-37016-5

**Published:** 2018-12-21

**Authors:** B. Majhy, R. Iqbal, A. K. Sen

**Affiliations:** Department of Mechanical Engineering, Indian Institute of Technology Madras, Chennai, 600036 India

## Abstract

We report a simple, inexpensive and rapid method for fabrication of a stable and transparent superhydrophobic (TSHB) surface and its reversible transition to a transparent superhydrophilic (TSHL) surface. We provide a mechanistic understanding of the superhydrophobicity and superhydrophilicity and the reversible transition. The proposed TSHB surface was created by candle sooting a partially cured n-hexane + PDMS surface followed by washing with DI water. The nano/microscopic grooved structures created on the surface conforms Cassie – Baxter state and thus gives rise to superhydrophobicity (water contact angle (WCA) = 161° ± 1°). The TSHB surface when subjected to oxygen plasma develops -OH bonds on the surface thus gets transformed into a TSHL surface (WCA < 1°). Both surface chemistry and surface morphology play important roles for the superhydrophobic to superhydrophilic transition. In the Cassie – Baxter relation for a composite surface, due to the capillary spreading of liquid in the nano/micro grooves, both *θ*_1_, *θ*_2_ = 0, thus giving rise to complete wetting. Rapid recovery of superhydrophobicity from superhydrophilicity was achieved by heating the TSHL surface at 150 °C for 30 min, due to a much faster adsorption of the -OH bonds into the PDMS. Thus it is possible to achieve reversible transition from TSHB to TSHL and vice versa by exposing to oxygen plasma and heat, respectively.

## Introduction

Fabrication of superhydrophobic and superhydrophilic surfaces has drawn significant attention in recent times due to innumerable applications of such surfaces in energy, water, health care, fundamental research as well as day-to-day activities^1^. Some of the specific applications of such extreme wetting surfaces include oil – water separation^2,3^, fog harvesting^4^, waste water treatment, lab on a chip device^5^, self – cleaning^6^, blood – plasma separation^7^ and cell biology^8–12^. Wetting behaviour of these surfaces is mainly governed by the microstructure (roughness) and chemical composition of the surfaces that requires deep fundamental understanding^13^.

The contact angle of a liquid on a smooth and chemically homogeneous solid surface is calculated by the Young’s equation given as $cos\theta =({\gamma }_{sg}-{\gamma }_{sl})/{\gamma }_{lg}$,where *θ* is Young’s contact angle, *γ*_*sg*_, *γ*_*sl*_ and *γ*_*lg*_ are solid-gas, solid-liquid and liquid-gas interfacial tension values. According to Girifalco – Good^14^, a maximum contact angle of 120° was achieved on a flat solid surface by lowering the surface energy up to $6.7\,{\rm{mJ}}/{{\rm{m}}}^{2}$, by using *CF*_3_ groups having closest hexagonal pack. In order to achieve extreme contact angle using rough surfaces, Wenzel proposed a model^15^, which is given as $cos{\theta }_{w}=rcos\theta$, where *r* is the roughness ratio (i.e. ratio of actual surface area to the apparent surface, *r* > 1), and *θ*_*w*_ is the Wenzel’s contact angle. According to this model, a hydrophobic surface becomes more hydrophobic and a hydrophilic surface becomes more hydrophilic by simply modifying the roughness ratio *r*. According to Johnson and Dettre^16^, contact angle *θ* and its hysteresis increases with roughness *r* on hydrophobic rough surfaces, in a regime where Wenzel state is dominant. From the equation, ${r}_{c}=1+(ta{n}^{2}\theta /4)$, contact angle *θ* continues to increase but hysteresis decreases with roughness for *r* > 1.75, because of transition from Wenzel state to Cassi-Baxter state due to entrapment of air in the grooves. According to Cassie-Baxter model, contact angle on a solid-air composite surface $cos{\theta }_{CB}=f(cos\theta +1)-1$, where *f* is the fraction of solid surface and *θ*_*CB*_ is the Cassie-Baxter angle. According to this equation, perfect non-wetting i.e. 180° contact angle, is not possible, since the fraction of solid *f* cannot be zero or *θ* = 180° is not possible. Also, it is difficult to achieve a contact angle beyond ${\theta }_{CB}=162^\circ$ (with $\theta =120^\circ \,\mathrm{and}\,\,f=0.1)$ ^17^ although Kao *et al*.^18^ have developed a fractal surface using alkylketene dimer (AKD) which showed a contact angle of 174°.

If the contact angle of water in air on a surface is >150°, it is referred as superhydrophobic surface. These surfaces are ubiquitous in nature as lotus leaf due to papillose epidermal cells covered by hydrophobic epicuticular wax^19^. These surfaces have a wealth of applications starting from commercial to biomedical applications. Superhydrophobic surfaces exhibit special properties due to which these surfaces are anti-corrosive, anti-icing, anti-drag and anti-sticking. Some of the applications of superhydrophobic surfaces include but not limited to hydrodynamic drag reduction^20,21^, self-cleaning^22^, condensation enhancement^23^, evaporation reduction^23^ and blood repellency^24^.

If the contact angle of water on a surface, in air, is <5°, it is known as superhydrophilic surface^13^. According to the Wenzel model, the minimum roughness required for complete wetting is, $r\ge \frac{1}{cos\theta }$. According to the equation, for *r* = 1.2-2, superhydrophilicity can be achieved if the contact angle of the initial smooth surface is <60°. But, if the contact angle >60°, it is difficult to enhance wettability by simply increasing the roughness^25^. In case of a composite surface, having solid and liquid fractions underneath a liquid drop^17^, with solid contact angle *θ*_1_ = *θ* and liquid contact angle *θ*_2_ = *θ*, the Cassie-Baxter relation is expressed as $cos{\theta }_{CB}=1-f+fcos\theta$. From this equation, we can conclude that it is not possible to achieve complete wetting $({\theta }_{CB}=0)$ by simply modifying the surface roughness^17^. Until now, several techniques, such as calcination^26,27^, sol-gel self-assembly^13^, spray coating, layer-by-layer assembly^17^, electrochemical anodization^28^, electrospinning, solution coating, etching, interfacial polymerization and hydrothermal treatment, have been developed for the fabrication of superhydrophilic surfaces by changing the surface chemistry to reduce surface energy and topography for increasing roughness. Similarly, different techniques have been developed for converting superhydrophobic surfaces to superhydrophilic surfaces, such as electrochemical alteration of the oxidation^29^, graphene interface^30^, nano-coating^31^, UV exposure^32^, phase separation and vapour deposition^33^. Recently, fabrication and characterization of a PDMS – derived candle soot coated superhydrophobic surface was reported^34^. However, the surface was not very stable and opaque (i.e. black in colour) due to the presence of carbon soot particles firmly embedded to the surface. A transparent, robust and superamphiphobic coating with candle soot as a template was fabricated by Deng *et al*.^35^.

A comprehensive review of the literature indicates that a mechanistic understanding of superhydrophobic to superhydrophilic transition and its reversal is missing. In literature, although there is some illustration of the effect of nano/microscopic roughness on extreme non-wetting (superhydrophobicity), there is no description on its effect on the extreme wetting (superhydrophilicity) and its reversible transition. Moreover, the role of precursor (capillary) contact line for superhydrophilicity of the rough surface has not been explained earlier, which is detailed in the present work. Here, we report a simple, inexpensive, rapid and one-step method for the fabrication of a stable and transparent superhydrophobic surface and its reversible transition to a superhydrophilic surface. We have used exposure of the surface to O_2_ plasma for the superhydrophobic-superhydrophillic transition and employed heating of the surface for the reverse transition. We have illustrated a detailed understanding of the surface chemistry and show that both roughness and surface energy are responsible for the extreme wetting (superhydrophilic) and non-wetting (superhydrophobic) conditions. The proposed superhydrophobic and superhydrophilic surfaces exhibit very good chemical and mechanical stability and are biocompatible. In contrast to the opaque superhydrophobic surface we reported earlier^34^, in the present case, the loosely attached carbon soot particles get washed away when exposed to high speed water jet thus leaving micro/nanoscopic surface asperities in the PDMS substrate that gives rise to the superhydrophobicity with water contact angle $({\rm{WCA}})=161^\circ \pm 1^\circ$.

In contrast to more complex and expensive approaches followed in the literature^29–34^, we demonstrate a simple and cost-effective approach to convert a superhydrophobic surface into a superhydrophilic surface. The superhydrophobic surface thus prepared when subjected to oxygen plasma^36^ develops -OH bonds on the surface thus gets transformed into a superhydrophilic surface (with WCA < 1°). The surface regains its superhydrophobic nature upon heating it at 150 *°C* for 30 min. First, we illustrate the science behind fabrication of a transparent and stable superhydrophobic surface. Then, we illustrate the science behind the reversible transition of the superhydrophobic surface to a superhydrophilic surface. Here, in the Cassie-Baxter relation, *f* → 0 and moreover, due to capillary spreading of liquid in the micro/nano grooves, both *θ*_1_, *θ*_2_ = 0, which gives rise to complete wetting. The effect of plasma exposure condition on superhydrophilicity is reported. The reversible transition^37–41^ cycles between superhydrophobicity and superhydrophilicity is also demonstrated.

## Fabrication of Transparent superhydrophobic (TSHB) and Transparent superhydrophilic (TSHL) surfaces

**Figure 1:**
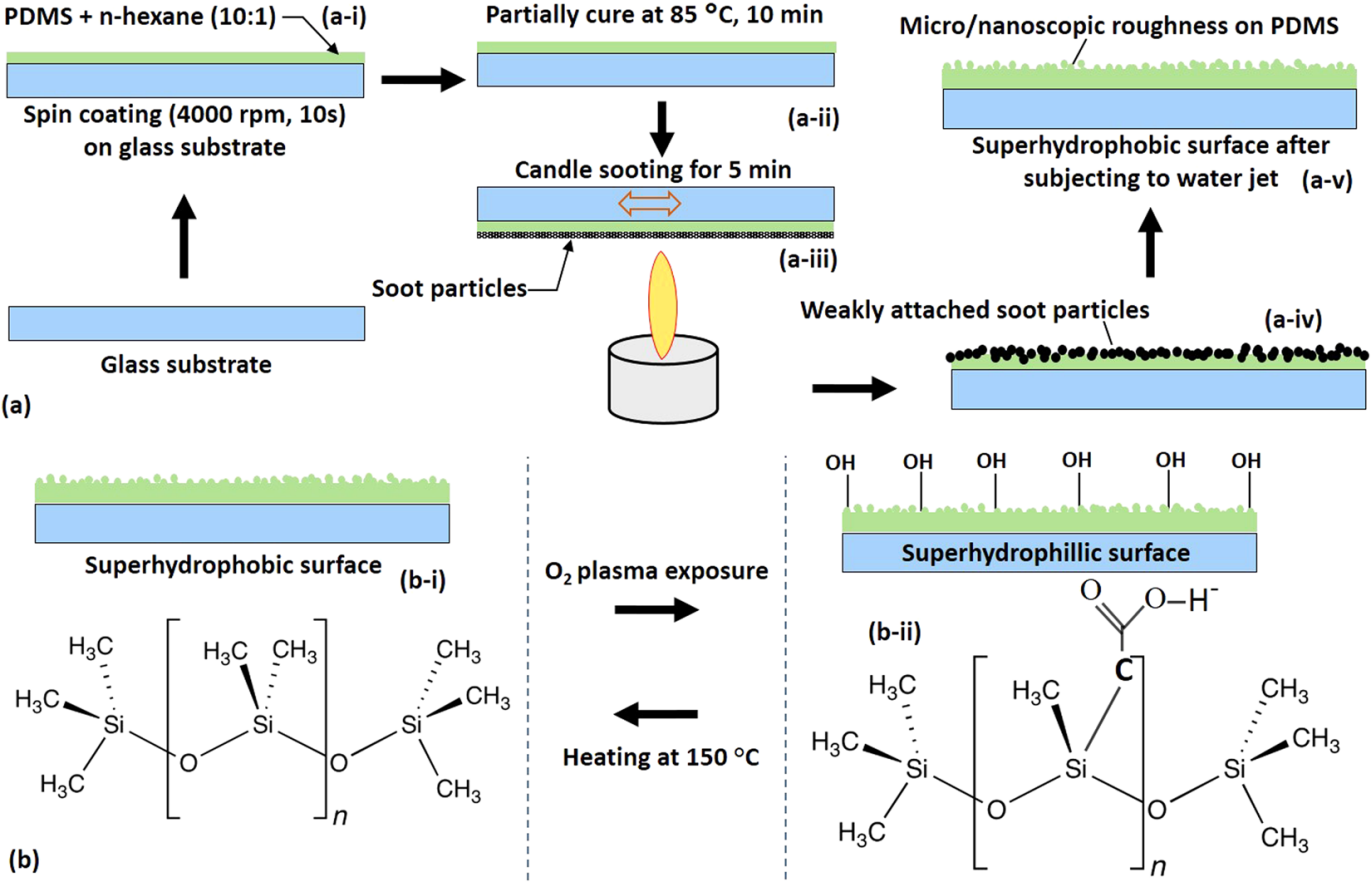
Schematic showing (**a)** fabrication of the stable and transparent superhydrophobic surface (**b**) reversible transition of a superhydrophobic surface to a superhydrophilic surface.

Firstly, PDMS base and curing agent at 10:1 ratio were mixed in a container and then the mixture was subsequently degassed in a desiccator until all the air bubbles are removed. Then, 10% of n-hexane was thoroughly mixed with the PDMS and kept in air for some time until all the air bubbles were removed (referred as “PDMS mixture” hereon). The purpose of adding n-hexane is to control the viscosity of the PDMS mixture to facilitate spin coating and embedding of soot particles into the PDMS layer, which is studied in detail in our previous work. Then, the glass slides, cleaned with IPA (isopropyl alcohol) in an ultrasonicator followed by spraying of high pressure N_2_ gas for drying, were coated with a 243 μm thickness (measured using an optical profilometer, Bruker, U.S.A.) of PDMS mixture at 4000 rpm for 10 s using a spin coater. Next, the coated layer of the PDMS mixture was partially cured at 85 °C for 10 min on a hot plate. The partially cured PDMS layers were then brought over the (centre of the) candle flame (upside down, see Fig. 1a) and moved across back and forth at a constant speed (in the same horizontal plane) for 5 min to obtain uniform layer of soot deposition. Next, the carbon soot coated glass slide was subjected to high speed water jet so that the weakly bonded (due to partial curing of the PDMS layer) soot particles are removed from the surface. The soot particles detached from the surface leave micro/nanoscopic rough structures on the surface of the PDMS, which gives rise to a transparent superhydrophobic (TSHB) surface (see Fig. 1a-iv).

The transparent superhydrophobic (i.e. PDMS with micro/nano-roughness) surface was exposed to oxygen plasma (PDC - 002, Harrick Plasma, U.S.A.) for different durations (0–5 min) at different power levels (10–30 W) to obtain the superhydrophilic surface. Exposure of the superhydrophobic surface to oxygen plasma produces -OH bonds on the surface which along with the micro/nano roughness is responsible for the superhydrophilicity. The transparent superhydrophilic (TSHL) surface thus prepared by exposing 30 W plasma for 2 min was heated at 150 °C for 30 min to convert it back to a superhydrophobic surface (see Fig. 1b). Heating of the superhydrophillic surface leads to faster adsorption of the -OH bonds into the bulk of the PDMS layer and finally, absence of any -OH bonds on the surface, thus giving rise to its native superhydrophobic state, rapidly.

## Results and Discussion

### Transparent Superhydrophobic (TSHB) Surface

**Figure 2:**
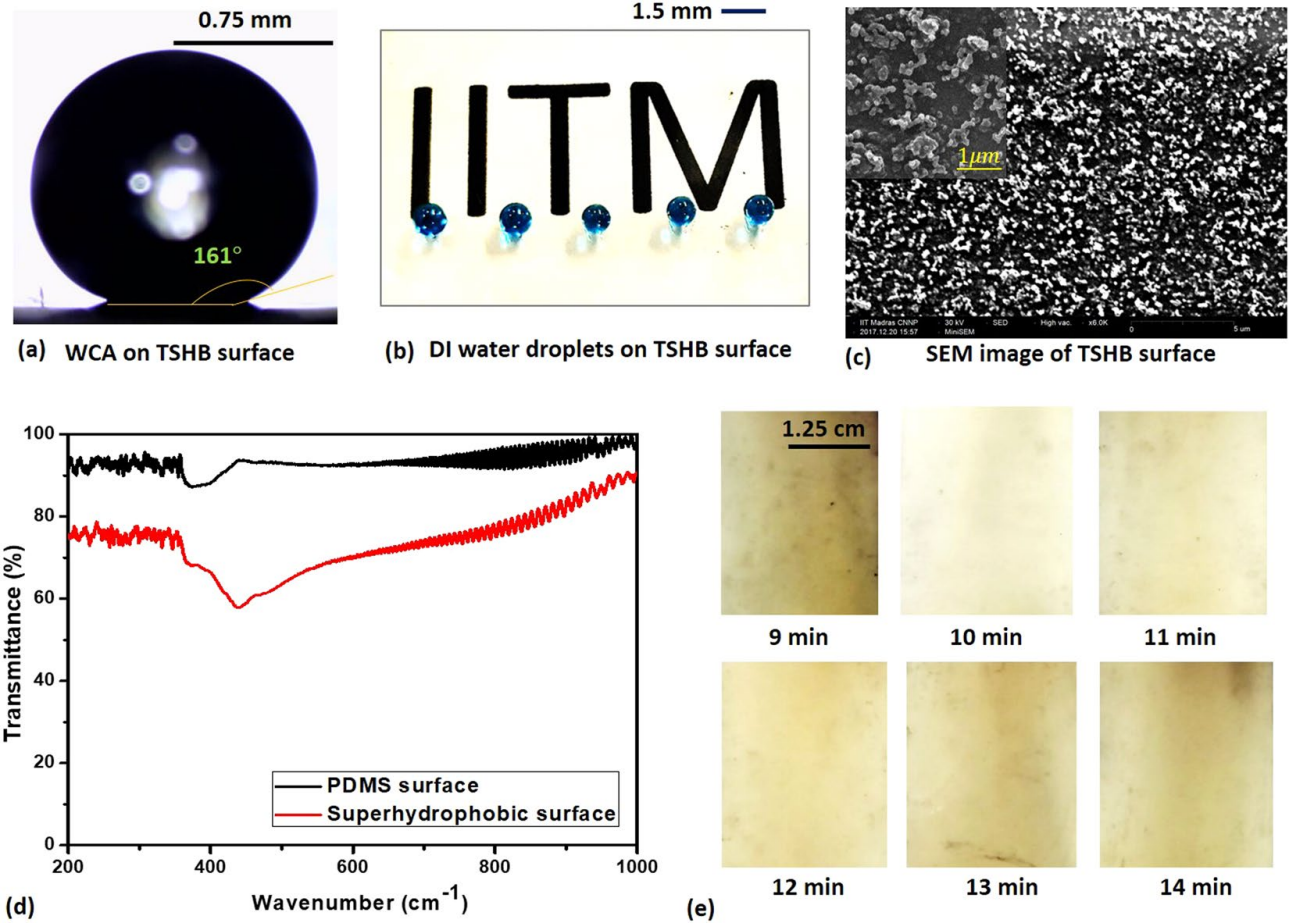
(**a**) Experimental image of a DI water droplet (of volume 5 µl) on the transparent superhydrophobic surface (**b**) Photograph of a transparent superhydrophobic surface (**c**) SEM image of the microstructure of the SHB surface (**d**) Transmittance of the superhydrophobic and PDMS surfaces (**e**) Transparency of the SHB surface with (partial) curing time.

The experimental image of a DI water droplet (of volume 5 µl) on the transparent superhydrophobic (TSHB) surface is shown in Fig. 2a, in which case the WCA was measured to be 1.61° ± 1°. The photograph of a TSHB surface fabricated using the procedure outlined in section 2 is shown in Fig. 2b. A thin layer of carbon particles^42^ gets weakly attached to the surface of the partially cured PDMS during the sooting process, which typically happens within 1.0 min. However, since the sooting process is carried out for a duration of 5 min, during this additional sooting time, some carbon particles get oxidized and removed from the surface due to convection. The additional unbound and weakly bound soot particles get removed during washing with high-speed water jet to yield the transparent superhydrophobic surface. The surface morphology of the TSHB surface was obtained by using scanning electron microscopy (SEM, HRSEM, Inspect F50, FEI). Figure 2c shows images of the surface microstructure. It is observed that nanometric size (~100 nm) rough grooved structures are formed on the surface that are comparable in size with that of the soot particles that leave the soot-coated PDMS surface during oxidation or washing with high-speed water jet. It is also observed that the size and structure, as well as the distribution of these grooves are very irregular. The irregular asperities consisting of nanometer-sized grooves in PDMS^43^ substrate give rise to the Cassie−Baxter state by increasing the effective roughness of the surface.

The transparency of the superhydrophobic surface is compared with that of a planar PDMS surface (coated on a similar glass slide) and the results are shown in Fig. 2d. It is observed that the transparency of the superhydrophobic surface is between 60 to 90% for wave numbers ranging between 200 to 1000 cm^−1^, which is comparable with that of a PDMS surface of 93%. The transparency of the TSHB surface was found to be dependent on the (partial) curing time, as depicted in Fig. 2e. It is observed that the transparency is maximum for a critical curing time of 10 min. If the curing time is shorter than the critical curing time, since PDMS does not get sufficiently cured, the soot particles penetrate deep and get firmly attached to the PDMS surface. Thus after spraying with water jet, some traces of carbon soot particles are still found on the PDMS surface which affects the transmittance. The soot particles remaining on the surface, apart from the transparency, would also affect the wettability of the substrate. Soot particles being hydrophobic in nature, with the increase in the remaining soot particles, the superhydrophobicity of the substrate would increase. But the same would have an adverse effect on the superhydrophilicity of the substrate. With more remaining soot particles, the substrate would not get completely exposed to O_2_ plasma resulting in lesser quantity of -OH bonds, which is directly related to the superhydrophilicity of the substrate. As the sooting time (5 min) is kept fixed in all cases, for a curing time longer than the critical curing time (i.e. 10 min), PDMS gets exposed to a high temperature for a longer duration which affects the transmittance. As observed, the surface appears more yellowish with increase in the curing time above the critical curing time.

### Mechanical and chemical stability of the TSHB surface

**Figure 3:**
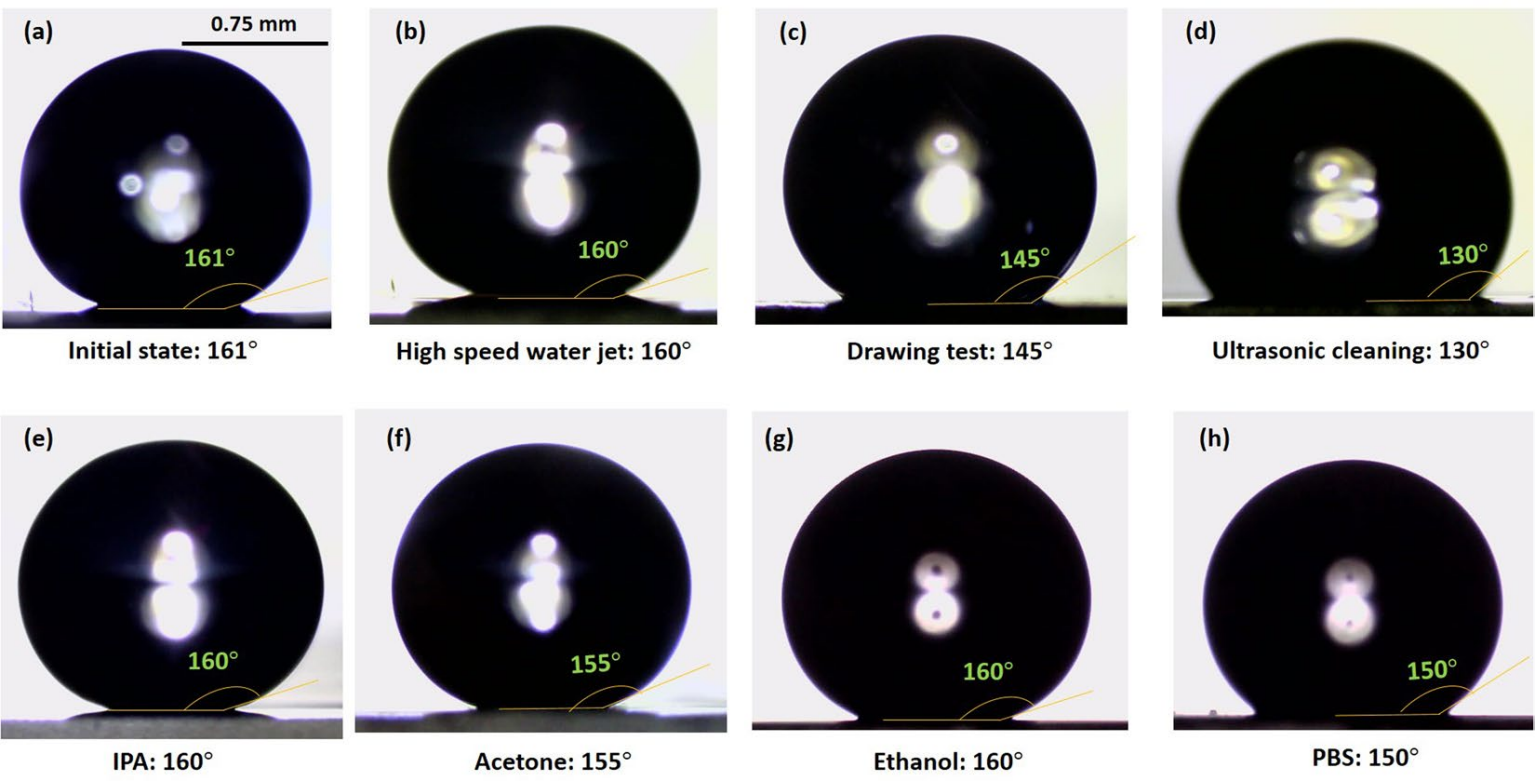
Water contact angle (WCA) of transparent superhydrophobic (TSHB) surface after different mechanical and chemical stability tests: (**a**) initial state; (**b**) high speed water jet impingement; (**c**) drawing test (**d**) ultra-sonication; (**e**) IPA dip; (**f**) acetone dip; (**g**) ethanol dip; (**h**) PBS dip.

In order to test the mechanical stability of the TSHB surface (Fig. 3a), the surface was subjected to high-speed (8 ms) water jet for a duration of 10 min, as shown in Fig. S5. The pressure imposed on the TSHB surface due to the impact of water was ~30lPa The WCA on the surface after the water-jet test was measured to be 160° ± 1° (see Fig. 3b) indicating the mechanical stability of the TSHB surface. Next, drawing test was performed to demonstrate the mechanical stability of the TSHB surface. The TSHB surface was drawn repeatedly against a polyester wipe (Essentra Wipes, China) under a pressure of 1.75 kPa (using a dead weight of 30 g) applied normal to the surface. The WCA of the surface after the drawing test was measured to be 450° ± 1° (see Fig. 3c), which showed less than 10% change in the WCA as compared to that before the drawing test. The superhydrophobic surface was then sonicated in an ultrasonicator (MX100QTD-3L, Maxsell, China) for 2 min and the WCA measured after the sonication was found to be 130° ± 1° (shown in Fig. 3d). The slight decrease in the WCA and hence hydrophobicity after the drawing test and sonication is attributed to the removal of any fragile rough structures at the PDMS surface.

The chemical stability of the TSHB surface was tested by dipping the surface in various solvents such as isopropyl alcohol (IPA) (Fisher Scientific), acetone (Fisher Scientific), ethanol and phosphate buffer solution (PBS) (0.01 M concentration, Sigma-Aldrich) for a duration of 10 min. The WCA of TSHB surfaces dipped in IPA, acetone, ethanol and PBS were measured to be 160° ± 1°, 150° ± 1°, 160° ± 1° and 150° ± 1°, respectively (shown in Fig. 3e–h). It was observed that the WCA remains unchanged in the case when the SH surface is dipped in IPA and ethanol and changes slightly (<7%) in case of acetone and PBS, thus indicating chemical stability of the TSHB surface. Due to salt particles covering the surface, there is a 10° WCA degradation after dipping in PBS and fine cracks were observed on the TSHB surface after acetone dipping, which causes WCA degradation by 5°. There was no notable change in the WCA after dipping the TSHB surface for 1 h in chemicals such as benzoic acid $({{\rm{C}}}_{6}{{\rm{H}}}_{5}{\rm{COOH}})$ and acetic acid (${{\rm{CH}}}_{3}{\rm{COOH}})$, which indicates that the surface can be used in different chemical environments.

### Superhydrophobic to superhydrophilic transition

**Figure 4:**
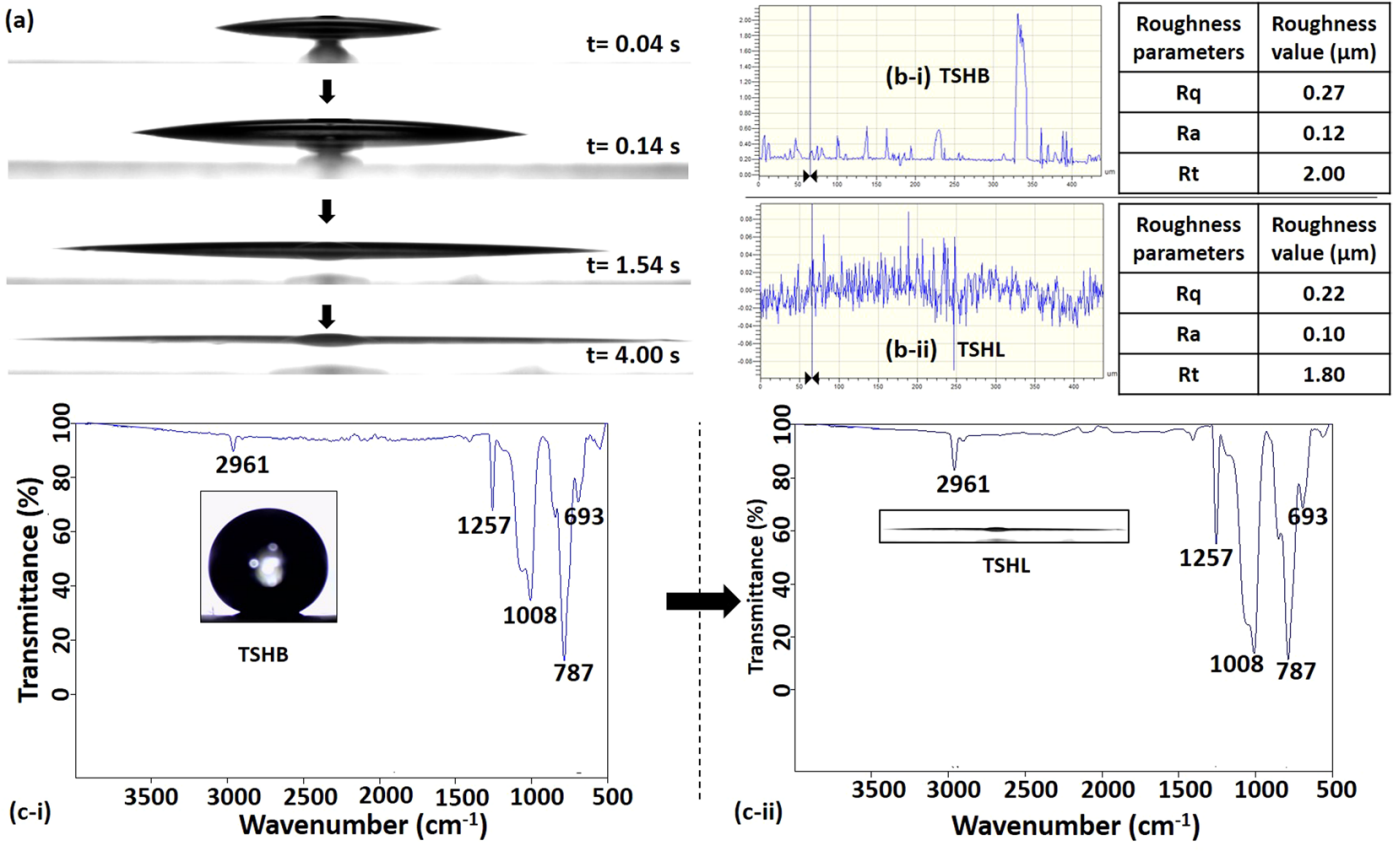
**(a**) Spreading dynamics (i.e. at different time instants) of a droplet of volume 5 µl on the TSHL surface. (**b)** Profilometry data showing roughness values of the TSHB and TSHL surfaces. (**c**) FTIR spectra showing the chemical compositions of the TSHB and TSHL surfaces.

The WCA due to a droplet of volume 5 µl dispensed on the TSHB surface was measured to be 161° ± 1° (see in Fig. 3a). Upon exposure of the surface to oxygen plasma^44,45^ for 2 min at 30 W power, the surface becomes superhydrophilic and the WCA of the droplet on the transparent superhydrophilic (TSHL) surface was measured to be <1°, as shown in Fig. 4a (also see Video S1). The spreading dynamics (i.e. at different time instants) of a droplet of volume 5 µl on the TSHL surface is shown in Fig. 4a and the variation of the contact line diameter with time is presented in Fig. S1 in the supporting information. Similarly, the spreading of an oil droplet of volume 5 µl is depicted in Fig. S2 which shows that the surface is also superoleophillic. The TSHL surface if more superoleophillic compared to the TSHB surface, as shown in Fig. S2.

The surface topography and nanoscale asperities of the TSHB and TSHL surfaces were characterized using profilometry and Fourier-transform infrared spectroscopy (FTIR) analysis. Figure 4b shows the profilometry images and roughness values of the TSHB and TSHL surfaces. The roughness values of the TSHB surface obtained from the measurements are as follows: average roughness (Ra) = 70 nm, root mean squared roughness (Rq) = 110 nm. Similarly, the roughness values of the TSHL surface are as follows: Ra = 60 nm, Rq = 90 nm. The data shows that there is a 15% reduction in the surface roughness, which is possibly due to removal of some of the fragile particles on the TSHB surface by high energy plasma. However, the roughness values show that both the surfaces are very rough, which gives rise to a stable Cassie−Baxter state favouring extreme wetting condition.

The chemical compositions of the TSHB and TSHL surfaces were studied using FTIR measurements, and the spectra are shown in Fig. 4c. For both surfaces, the various functional groups were identified and reported on the spectra. The following peaks are present in both the TSHB and TSHL surfaces. In the high frequency region, the adsorption peak located at $2961\,{{\rm{cm}}}^{-1}$ is attributed to the asymmetric stretching vibration of CH_3_ group, while the peak located at 1409 cm^−1^ is attributed to C-H deformation vibration. The peaks located at $844\,{{\rm{cm}}}^{-1}$ and $787\,{{\rm{cm}}}^{-1}$ represent CH_2_ rocking vibration and Si-C stretching in Si-CH_3_ and that at $694\,{{\rm{cm}}}^{-1}$ is due to CH_2_ deformation vibration. For both the TSHB and TSHL surfaces, the transmittance intensities corresponding to each of the above peaks were found to be the same. From the results, it is evident that the TSHB and TSHL surfaces are composed of a mixture of branched saturated hydrocarbons, methylene, and methyl groups. However, for the TSHL surface, the transmittance intensity is found to be significantly enhanced at the following peaks (as compared to TSHB surface). The peak located at wavenumber 1257 *cm*^−1^ corresponding to COOH (n-aliphatic) bond due to *O*-*CO* in-plane deformation vibration and peak at wavenumber 1008 *cm*^−1^ corresponds to SiOH bond. These bonds have polarizable ions, so water molecules create polarized bonds with the TSHL surface molecules and completely spread on the surface.

### Mechanism of superhydrophobic to superhydrophilic transition

**Figure 5:**
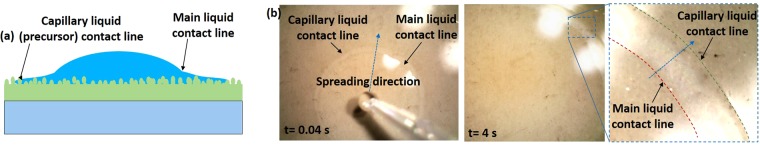
(**a**) Schematic of the mechanism of spreading of a liquid on TSHL surface. (**b**) Experimental images showing spreading of a DI water droplet of volume 5 µl on a TSHL surface.

Figure 5a shows a schematic of the spreading of a liquid (such as DI water or oil) on the TSHL surface. Figure 5b shows the experimental images of spreading of a DI water droplet of volume 5 µl on a TSHL surface (see Videos S2 and S3). Interestingly, two separate contact lines, namely main liquid contact line and capillary flow (precursor) contact line, are observed. The advancement of the main liquid contact line represents the spreading of the droplet on the surface due to the high surface energy. Due to the presence of nano/microscopic asperities on the TSHL surface, capillary flow of liquid in the nano/micro roughness structures (ahead of spreading of the main liquid droplet) is exhibited. Thus the surface roughness grooves and the solid in between are covered with a thin layer of liquid (see Fig. 5a,b). Considering the Cassie-Baxter relation, $cos{\theta }_{CB}=1-f+fcos\theta$, due to the capillary spreading of liquid in the micro/nano grooves ahead of the main droplet contact line, we have *θ* = 0, which gives rise to complete wetting condition (i.e. WCA of <1°). The variations in the main liquid droplet and precursor contact line with time are presented in Fig. S1. It is observed that the spacing between the main liquid contact line and precursor contact line increases with time. The main liquid line stops when the composite system attains a minimum energy state. The precursor contact line stops when the driving capillary force is balanced by the sum of the viscous force and vapour pressure (mass rate decrement due to evaporation). Capillary line is faster than spreading of droplet line because in case of capillary flow *x* ~ *t*^0.5^ according to Washburn, where as in case of droplet spreading^46^ *x* ~ *t*^0.3^, where *x* is meniscus position and *t* is time.

**Figure 6:**
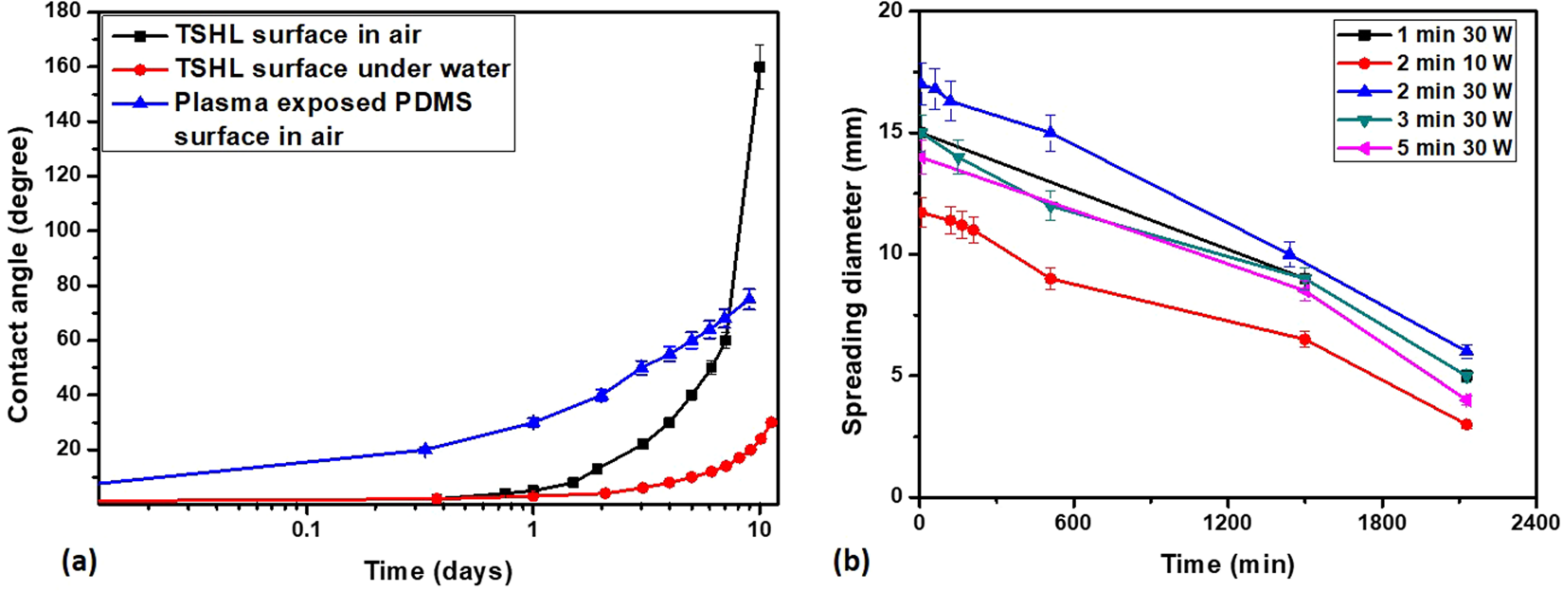
(**a**) Comparison of the variation of the wettability of the TSHL and plasma-exposed smooth PDMS surface with time in air and stability of the wettability of the TSHL surface when stored in DI water. (**b**) The variation of the equilibrium contact line diameter (CLD) with time for different plasma exposure time.

Both surface chemistry, which decides the surface energy, and surface morphology, which decides the surface roughness, play important roles for the superhydrophobic to superhydrophilic transition. To confirm this, a smooth PDMS surface was exposed to oxygen plasma at the same conditions and its wettability was compared with that of the proposed TSHL surface, as shown in Fig. 6a. Although, the plasma exposed smooth PDMS surface exhibits the same chemical composition as the superhydrophilic surface (i.e. with carboxyl (COOH) and SiOH bonds as shown in Fig. S3 in supporting information), the WCA was found to be higher than 5° ± 1°. In this case, no precursor contact line was observed (see Fig. S3 in supporting information), which is responsible for the superhydrophilicity.

Next, we discuss the effect of plasma exposure condition on the superhydrophilicity by measuring the final contact line diameter of a 5 µl DI water droplet dispensed on the TSHL surface. Figure 6b shows the variation of the equilibrium contact line diameter (CLD) with time for different plasma exposure times. The CLD was found to be highest for an exposure time of 2 min at 30 W power. For a longer exposure time (or power), the roughness of the surface decreases which lowers the capillary pressure driving the precursor flow thus giving rise to a smaller CLD. On the other hand, for a lower exposure time (or power), the number density of the carboxylic (COOH) and silane (SiOH) groups are lower thus offering a smaller CLD. Thus both roughness and chemical composition of the surface play important roles in deciding the wettability or spreading condition of the TSHL surface.

WCA on PDMS surface is 100° ± 0.5° and total surface energy is $22\pm 0.25\,mN\,{m}^{-1}$, which is a combination of $21\pm 0.15\,mN\,{m}^{-1}$ due to dispersion and $1\pm 0.15\,mN\,{m}^{-1}$ due to polarization, as measured by DSA25 (Kruss, Germany) goniometer using Owens, Wendt, Rabel and Kaelble (OWRK) method using water as polar liquid and di-iodomethane as disperse liquid (see Table 1). After exposing the PDMS surface with oxygen plasma for 2 min at 30 W power, WCA decreases to 5° ± 0.1° due to increase in the total surface energy to $76\pm 0.25\,{\rm{mN}}\,{{\rm{m}}}^{-1}$. The increase in surface energy is mainly attributed to the formation of polarized ions siloxane (Si-OH) and carboxyl (COOH) formed at the surface of PDMS due to plasma exposure (see FTIR analysis shown in Fig. S3a in supporting information). The surface energy of the superhydrophobic surface is $7.85\pm 0.25\,mN\,{m}^{-1}$ as measured by DSA25 using OWRK method. But according to OWRK method, which is given as $\frac{{\gamma }_{L}(cos\theta +1)}{2{({\gamma }_{L}^{D})}^{\frac{1}{2}}}={({\gamma }_{S}^{P})}^{\frac{1}{2}}[\frac{{({\gamma }_{L}^{P})}^{\frac{1}{2}}}{{({\gamma }_{L}^{D})}^{\frac{1}{2}}}]+{({\gamma }_{S}^{D})}^{\frac{1}{2}}$, where *γ*_*S*_ and *γ*_*L*_ are surface energy of solid and liquid respectively. We see that WCA is a function of surface energy alone since the method does not consider roughness ratio (*r*) for calculating ${\gamma }_{S}={\gamma }_{S}^{P}+{\gamma }_{S}^{D}$. For the TSHB surface, by using Cassie – Baxter relation $cos{\theta }_{C-B}=f(rcos\theta +1)-1$ and values of ${\theta }_{C-B}=161^\circ ,\,f=0.1\,{\rm{and}}\,\theta =100^\circ$, we calculate that the roughness ratio *r* = 2.8. Surface energy of smooth PDMS surface $22\pm 0.25\,{\rm{mN}}\,{{\rm{m}}}^{-1}$ to surface energy of TSHB surface $7.85\pm 0.25\,{\rm{mN}}\,{{\rm{m}}}^{-1}$ by OWRK method is a factor of 2.8, which is same as roughness ratio *r* = 2.8. Thus surface energy of the TSHB surface is roughness ratio -times the surface energy of the PDMS surface and similarly, the surface energy of the TSHL surface is roughness ratio -times the surface energy of plasma exposed PDMS surface, as shown in the table below. Thus it is confirmed that both roughness and surface energy are responsible for the extreme wetting (superhydrophilic) and non-wetting (superhydrophobic) conditions.

**Table 1:** Surface energy and WCA of different surfaces.

	Water contact angle (deg)	*γ*_*s*_ (mN m^−1^)	${{\boldsymbol{\gamma }}}_{{\bf{s}}}^{{\bf{D}}}$ (mN m^−1^)	${{\boldsymbol{\gamma }}}_{{\bf{s}}}^{{\bf{P}}}$ (mN m^−1^)
PDMS	100°	22 ± 0.25	21 ± 0.15	1 ± 0.15
PDMS-Plasma	5°	76 ± 0.25	39.5 ± 0.15	36.5 ± 0.15
Superhydrophobic	161.5° ± 0.5°	22 ± 0.25	21 ± 0.15	1 ± 0.15
Superhydrophilic	1°	76 ± 0.25	39.5 ± 0.15	36.5 ± 0.15

### Stability and biocompatibility of the TSHL surface

Next, we study the stability of the wettability of TSHL surface and compare the same with that of a plasma exposed smooth PDMS surface, which is shown in Fig. 6a. Both the surfaces were exposed at the same condition i.e. for a duration of 2 min at 30 W power and then kept in ambient air. As observed, the WCA of the smooth PDMS surface increases much faster as compared to the TSHL surface, which is explained as follows. The transformation of the TSHL surface from superhydrophilicity to hydrophilicity, then to hydrophobicity and finally to superhydrophobicity is attributed to the adsorption of the -OH bonds into the bulk of the PDMS layer. In case of the TSHL surface, due to the presence of higher density of -OH bonds, it takes a much longer time to complete the adsorption of the surface bonds into the bulk as compared to the smooth PDMS surface. From Fig. 6a, we can see that PDMS takes only <8 h for the WCA to change form 5° ± 1° to 20° ± 1°, whereas the TSHL surface takes >48 h for the WCA to change from <1° to 10° ± 1°. Further, the wettability of the TSHL surface was significantly improved by storing it inside DI water, as shown in Fig. 6a. It is observed that the superhydrophilicity is retained for >15 days, which could be explained by considering that the high adhesive force and ions present in water prevent adsorption of surface -OH bonds into the bulk material. On the other hand, due to absence of ions and low adhesive force, superhydrophilicity is retained only for 2 days, when kept in ambient air.

To demonstrate biocompatibility of the TSHB/TSHL surface, the TSHB surface was exposed to oxygen plasma at 10 W power for 30 s and HeLa cells were cultured using the protocol mentioned in section 2. Figure S4 in supporting information shows growth of cells after 48 h indicating that the proposed TSHB/TSHL surface is biocompatible.

### Reversible superhydrophobic-superhydrophilic transition

**Figure 7:**
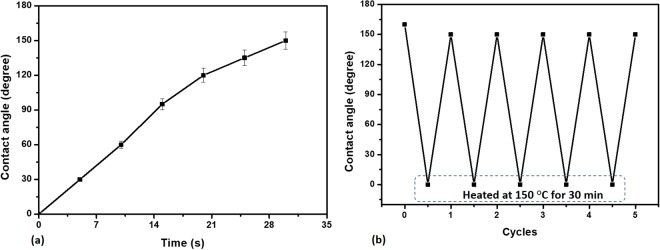
(**a**) Rapid transition of a TSHB surface to a TSHL surface upon exposure to plasma and its reversibility upon heating at 150 °C for 30 min. (**b**) Wetting transition cycles upon plasma exposure and heating.

As discussed, instant transition of superhydrophobicity to superhydrophilicity can be achieved by exposure of the TSHB surface to oxygen plasma (see Fig. 7a). Similarly, rapid recovery of superhydrophobicity from superhydrophilicity was achieved by heating the TSHL surface at 150 °C for 30 min. Heating of the superhydrophilic surface leads to a much faster adsorption of the -OH bonds into the bulk of the PDMS layer and finally absence of any -OH bonds on the surface, thus giving rise to its native superhydrophobic state, rapidly, as shown in Fig. 7a. Thus it is possible to achieve reversible transition between superhydrophobic to superhydrophilic by exposing to oxygen plasma and heat, respectively. We have carried out wetting transition cycles several times (see Fig. 7b) which show that the surface can be tuned between superhydrophobicity and superhydrophilicity, repeatedly. Moreover, the wetting transition process is achieved rapidly with a duration of 35 min for each cycle.

### Effect of roughness on superhydrophilicity and its reversible transition

**Figure 8:**
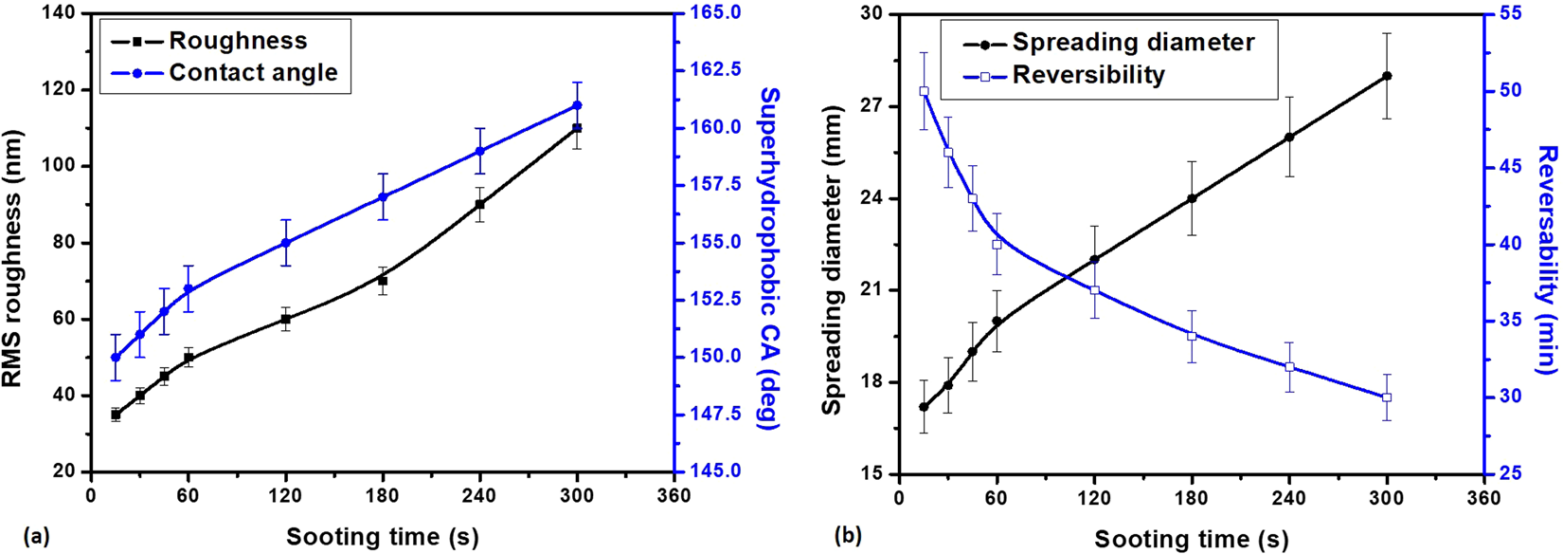
Effect of sooting time on (**a**) surface roughness and contact angle, (**b**) superhydrophilicity and heating time (at 150 *°C*) required for the superhydrophilic to superhydrophobic transition.

In order to investigate the effect of the roughness on the wettability of the substrate, we have varied the sooting time and obtained roughness values ranging from 35 to110 nm. The variation of roughness and the contact angle with sooting time is shown in Fig. 8a. We observed, with the increase in the sooting time, the roughness increases owing to more number of soot particles coming in contact with the substrate as well as due to the higher depth-wise penetration of the soot particles into the semi-cured PDMS substrate. With the enhanced roughness, the superhydrophobicity of the substrate also increases due to the entrapped air in the micro/nanoscopic grooves. The effect of the roughness on the superhydrophilicity (upon exposure of the TSHB surface to O_2_ plasma) and its reversible transition is shown in Fig. 8b. The variation in the superhydrophilicity is shown in terms of spreading diameter as it is very challenging to measure contact angle, which is very low (~0^*o*^). We observed, with increase in the roughness, the spreading diameter increases, which indirectly corroborates enhanced wetting. With an increase in roughness and covering of the grooves with a thin layer of water, the contact between main liquid line and the solid surface deceases thus superhydrophilicity increases. The effect of roughness on the reversible transition is shown in Fig. 8b, which suggests that, with increase in roughness, the heating time required for the transition decreases. This may be because; at higher roughness, heat transfer is enhanced thus facilitating faster adsorption of -OH groups into the bulk of the substrate.

## Conclusions

Here, we reported a simple, inexpensive, rapid and one-step method for the fabrication of a stable and transparent superhydrophobic (TSHB) surface and its reversible transition to a transparent superhydrophilic (TSHL) surface. The proposed TSHB surface was created by candle sooting a partially cured n-hexane + PDMS surface followed by washing with DI water. The mechanical and chemical stability and biocompatibility of the surface was demonstrated. We also provided a mechanistic understanding of the superhydrophobic and superhydrophilic conditions and the reversible transition. The nano/microscopic grooves present on the surface conforms to the Cassie – Baxter state and thus giving rise to superhydrophobicity (WCA = 161° ± 1°). The TSHB surface thus prepared when subjected to oxygen plasma (for 2 min at 30 W power) develops -OH bonds on the surface thus gets transformed into a TSHL surface (WCA < 1°). Both surface chemistry, which decides the surface energy, and surface morphology, which decides the surface roughness, play crucial roles for the superhydrophobic to superhydrophilic transition. Due to the presence of nano/microscopic asperities on the TSHL surface, capillary (precursor) flow of liquid over the nano/micro roughness structures (ahead of spreading of the main liquid droplet) is exhibited. In the Cassie – Baxter relation for a composite surface,$\,cos{\theta }_{CB}=1-f+fcos\theta$, due to the capillary flow of precursor liquid in the micro/nano grooves, *θ* = 0, thus giving rise to complete wetting. Rapid transition of superhydrophobicity from superhydrophilicity was achieved by heating the TSHL surface at 150 °C for 30 min, due to a much faster adsorption of the -OH bonds into the PDMS substrate. Thus it is possible to achieve reversible transition between TSHB and TSHL surfaces by exposing the TSHB and TSHL surfaces. In conclusion, the proposed TSHB and TSHL transition would motivate further research towards interesting applications.

## Materials and Methods

### Materials

PDMS (polydimethylsiloxane) silicone elastomer (base) and curing agent (Sylgard 184, Dow Corning, U.S.A.) and N-hexane (purity 99%, Spectrochem, India) were purchased for preparing the transparent superhydrophobic and superhydrophilic surfaces. Clean, transparent and superfine glass slides (Science House, Chennai, India) of thickness 1.1 mm were used as the base substrates for spin coating PDMS and candle sooting purposes. Paraffin wax candle of 4 cm diameter and 2 mm wick diameter was purchased from the local market for candle sooting. Deionized (DI) water of resistivity 18.2 MΩ·cm (DI water purification system, ELGA, U.K.) was used for characterizing the WCA on different wettable surfaces. Chemicals were used as received without any further modification/purification.

### Biocompatibility of the TSHB/TSHL surface

To demonstrate biocompatibility, a TSHB rectangular box (5 sides closed but top side opened) of size $7\times 2.5\times 2.5\,{{\rm{cm}}}^{3}$ was made using TSHB glass slides with the help of glue. The TSHB rectangular box was exposed to 10 W oxygen plasma for 30 s. Then the mild hydrophilic rectangular box made sterile using 70% ethanol spray. Cervical cancer cell line HeLa at concentration, 2 × 10^5^/mL was collected by trypsinization using Trypsin EDTA solution and centrifuged at 1500 rpm for 7 min. The collected cells were suspended in Dulbecco’s Modified Eagle Medium (DMEM) (Himedia, India) with Antibiotic Antimycotic solution (Himedia, India) and seeded into the mild hydrophilic rectangular box and then top of rectangular box was covered with a cleaned glass slide. The setup was placed inside the CO_2_ Incubator and cell growth have been monitored after 24 and 48 h. Finally, cell images have taken under Inverted microscope (IX 73 Olympus, Japan) and high-speed camera (FASTCAM SA5, Photron, UK).

## Supplementary information

Supplementary Information

video S1

video S2

video S3
