# Supplementary material for: Facile fabrication and mechanistic understanding of a transparent reversible superhydrophobic – superhydrophilic surface
Source: Sci Rep. 2018 Dec 21;8:18018. doi: 10.1038/s41598-018-37016-5 (PMC6303342; doi:10.1038/s41598-018-37016-5)
Supplement: Supplementary file 1 — Supplementary Information [file 41598_2018_37016_MOESM1_ESM.docx]

**Supplementary Information**

**Facile fabrication and mechanistic understanding of a transparent reversible superhydrophobic – superhydrophilic surface**

# B. Majhy, R. Iqbal and A. K. Sen^*^

Department of Mechanical Engineering, Indian Institute of Technology Madras, Chennai-600036, India.

^*^Author to whom correspondence should be addressed. Email: ashis@iitm.ac.in

**Fig. S1** The variation of the contact line diameter (both main contact line and precursor contact line) with time in case of a DI water droplet of 5 µl volume dispensed over a TSHL surface.

**Fig. S2** Spreading of a mineral oil droplet of volume 5µl on TSHB and TSHL surface.

**Fig. S3** (a) Chemical composition of the plasma exposed (30 W for 2 min) smooth PDMS surface, (b) Image showing spreading of a DI water droplet of 5 µl volume dispensed on the plasma exposed smooth PDMS surface.

**Fig. S4** Culture of HeLa cells on TSHB/TSHL surface (by exposing 10 W oxygen plasma for 30 secs on TSHB surface) (a) before cell culturing (b) after 48 hours of cell culturing.

**Fig. S5** (a) Schematic representation of mechanical stability test under high velocity (8 m/s) water jet. (b) Contact angle of water droplet on TSHB surface after high speed water jet impact.
